# Dietary Supplementation of Cedryl Acetate Ameliorates Adiposity and Improves Glucose Homeostasis in High-Fat Diet-Fed Mice

**DOI:** 10.3390/nu15040980

**Published:** 2023-02-16

**Authors:** Jingya Guo, Mengjie Li, Yuhan Zhao, Seong-Gook Kang, Kunlun Huang, Tao Tong

**Affiliations:** 1 Key Laboratory of Precision Nutrition and Food Quality, Key Laboratory of Functional Dairy, Ministry of Education, College of Food Science and Nutritional Engineering, China Agricultural University, Beijing 100083, China; 2 Department of Food Engineering, Mokpo National University, Muangun 58554, Republic of Korea; 3 Key Laboratory of Safety Assessment of Genetically Modified Organism (Food Safety), Ministry of Agriculture and Rural Affairs of the China, Beijing 100083, China; 4 Beijing Laboratory for Food Quality and Safety, Beijing 100083, China

**Keywords:** cedryl acetate, high-fat diet, obesity, gut microbiota

## Abstract

Cedryl acetate (CA), also called acetyl cedrene, is approved by the FDA as a flavoring or adjuvant to be added to foods. In this study, we aimed to investigate the preventive benefits of CA on obesity and obesity-related metabolic syndrome caused by a high-fat diet (HFD). Three groups of C57BL/6J mice (ten-week-old) were fed Chow, an HFD, or an HFD with CA supplementation (100 mg/kg) for 19 weeks. We observed that CA supplementation significantly reduced weight gain induced by an HFD, decreased the weight of the visceral fat pads, and prevented adipocyte hypertrophy in mice. Moreover, mice in the CA group showed significant improvements in hepatic lipid accumulation, glucose intolerance, insulin resistance, and gluconeogenesis compared with the mice in the HFD group. Since 16S rRNA analysis revealed that the gut microbiota in the CA and HFD groups were of similar compositions at the phylum and family levels, CA may have limited effects on gut microbiota in HFD-fed mice. The beneficial effects on the metabolic parameters of CA were reflected by CA’s regulation of metabolism-related gene expression in the liver (including *Pepck*, *G6Pase*, and *Fbp1*) and the epididymal white adipose tissues (including *PPARγ*, *C/EBPα*, *FABP4*, *FAS, Cytc*, *PGC-1α*, *PRDM16*, *Cidea*, and *COX4*) of the mice. In summary, a potent preventive effect of CA on HFD-induced obesity and related metabolic syndrome was highlighted by our results, and CA could be a promising dietary component for obesity intervention.

## 1. Introduction

Obesity refers to one of the multifactor-induced chronic metabolic diseases and is characterized by an increase in adipocytes (size and number) and an abnormal elevation of body fat percentage [1]. In 2020, the World Health Organization (WHO) reported that between 1975 and 2016, the global prevalence of obesity nearly tripled; in excess of 1.9 billion people aged ≥18 were overweight, while more than 650 million adults were obese [2]. Furthermore, obesity is an essential danger for many chronic diseases, such as nonalcoholic steatohepatitis, hypertension, muscle atrophy, and type 2 diabetes mellitus, which place an enormous burden on society [3,4]. Therefore, obesity has become not only an epidemic worldwide but also a major public health concern. During the past few decades, searching for new substances from foods, food ingredients, and natural products that are capable of preventing or treating obesity and related metabolic syndromes has been a popular research issue [5].

The gut microbiota is a complicated microbial community dwelling in the gastrointestinal tract and develops a close symbiotic relationship with its host [6,7]. To date, the gut microbiota has been recognized as being related to various physiological and pathological processes in the human body, which include preventing pathogen colonization, fermenting indigestible food components, synthesizing some vitamins, and contributing to the maturation of the immune system [8]. Notably, mounting evidence supports a connection between the dysbiosis of microbial ecology and metabolic diseases [9,10,11], particularly obesity [12,13]. Bäckhed et al. transplanted the microbiota of normally grown mice to germ-free mice, and the latter had a 60% increase in body fat content with reduced food intake, which provided pioneering evidence linking the gut microbiota with the progression of obesity [14]. In addition, certain natural products that have been reported to prevent or treat obesity are also capable of modifying gut microbiota, such as eugenol [15], Korean red ginseng [16], and pistachio [17].

**Figure 1:**
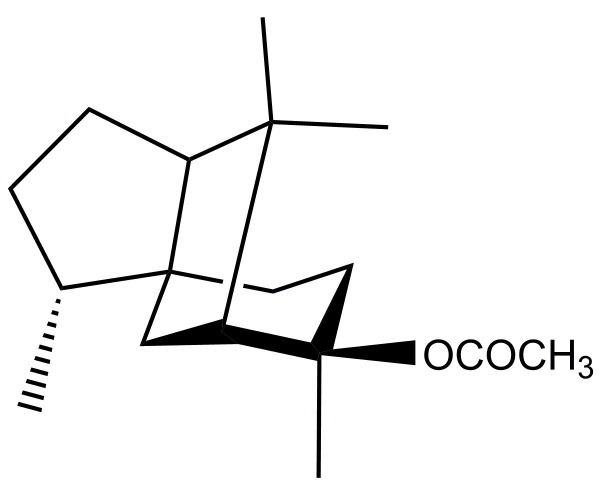
The chemical structure of CA.

Cedryl acetate (CA, Figure 1), also called acetyl cedrene, is a tricyclic sesquiterpene that naturally exists in the essential oils of *Platycladus orientalis* [18] and *Schinus molle L.* (false pepper) [19]. Since it has stable, long-lasting, and intense woody fragrances, CA is commonly used in producing cosmetics, soaps, perfumes, and other products with fragrances. Aside from the above roles of CA, it is worth noting that it has been approved by the U.S. Food and Drug Administration (FDA) for use as a flavoring or adjuvant in foods [20], and it is permitted as a spice for food according to China’s National Food Safety Standard (GB 29938-2020). At present, CA has been reported to have α-glucosidase inhibitory activity [21] and antifungal [22] activity. Our previous studies have revealed that oral administration of two analogs of CA, i.e., α-cedrene [23] and methyl cedryl ether [24], can prevent or reverse HFD-induced obesity and abnormal metabolic aberrations in rodents. Nevertheless, whether the consumption of CA affects obesity and related metabolic syndromes has not yet been elucidated.

Herein, we intended to assess the protective potential of dietary CA supplementation against high-fat diet (HFD)-induced obesity and explore whether the gut microbiota has a pronounced role in modifying the beneficial effects of CA against host adiposity.

## 2. Materials and Methods

### 2.1. Animals, Diets, and Design

Twenty-four 7-week-old C57BL/6J male mice were acquired from Vital River (Beijing, China). The animals were accommodated in the Animal Center (SYXK (Jing) 2020-0052 at 22 ± 2 °C, 40–70% relative humidity with a 12 h light to 12 h dark cycle). Mice were allocated into three groups (*n* = 8), i.e., Chow, HFD, and CA groups following three weeks of acclimatization. The standard Chow diet was provided by HFK Bioscience (Beijing, China, Supplementary Table S1), while the HFD (containing 40% fat) and HFD with supplementation of 0.1% CA (*w*/*w*) were obtained by following the previous description by Kim et al. [25]. The formulations of the HFD and HFD with CA supplementation are presented in Table 1. During the 19-week experiment, all the mice were fed their respective diets accompanied by weekly weighing. Access to food and water was freely available to all the mice.

At the end of the experiment, fresh stool samples were collected and stored immediately at −80 °C. Blood samples were obtained and stored in serum at −80 °C. The mice were then sacrificed by cervical dislocation. The perirenal, retroperitoneal, mesenteric, and epididymal white adipose tissue (eWAT) of the mice were taken out, weighed, and stored at −80 °C after immediate freezing in liquid nitrogen.

### 2.2. Dosage Information

The dose of CA (analytical reagent grade, Aladdin, Shanghai, China) for the mice in the study was 100 mg/kg of body weight, which is equivalent to 8.11 mg/kg of body weight in humans. The conversion calculations were estimated using the body surface area (BSA) normalization methodology as reported formerly by Reagan-Shaw et al. [26].

### 2.3. Oral Glucose Tolerance Test (OGTT) and Fasting Blood Glucose (FBG)

FBG testing was conducted on the mice with morning fasting (6 h, between 8:00 a.m. and 2:00 p.m.). The concentration of blood glucose was tested by a glucometer with blood glucose test strips, which were both obtained from Accu-Chek (Basel, Switzerland). On the following day, fasting was performed as described above; then, the OGTT was performed after gavaging the mice with D-(+)-glucose (2 g/kg body weight concentration, Sigma, St. Louis, MO, USA). The blood glucose concentration was measured before administration (recorded as 0 min), and the blood glucose concentrations were tested at 15, 30, 60, 90, and 120 min after administration; the area under the curve (AUC) was also calculated.

### 2.4. Insulin Tolerance Test (ITT)

Mice fasted as described in 2.3; ITT tests were performed after the mice were injected with insulin intraperitoneally. The concentration of insulin, purchased from Novo Nordisk (Copenhagen, Denmark), was 0.75 U/kg of body weight. The blood glucose measurement time was the same as described in 2.3.

### 2.5. Pyruvate Tolerance Test (PTT)

The PTT was performed by intraperitoneal injection of sodium pyruvate after 16 h of fasting (7:00 p.m. to 11:00 a.m.) in the mice. The concentration of sodium pyruvate, purchased from Blotopped (Beijing, China), was 1 g/kg of body weight. The blood glucose measurement time was the same as that stated in 2.3.

### 2.6. Serum Biochemical Analysis

Biochemical parameter kits were obtained from Bio-Technology and Science (Beijing, China). The concentrations of all biochemical parameters in our study were measured with a Thermo Fisher Indiko analyzer (Waltham, MA, USA).

### 2.7. Histopathological Analysis 

Tissues from the liver and eWAT were necropsy-dissected and fixed in a 4% paraformaldehyde solution for at least 24 h. After dehydrating with ethanol and embedding the tissue in paraffin, the staining of tissues with a thickness of 4 to 5 μm was performed by hematoxylin and eosin (H&E). Observation of histopathological changes was performed under a Leica DM750 microscope (Nussloch, Germany). The size of the adipocytes was calculated by Image J (version 1.53).

### 2.8. Gut Microbiota Analysis

DNA was isolated from the fecal matter produced by the mice. A Thermo Fisher Nano-300 microspectrophotometer (Waltham, MA, USA) was applied to measure the purity of the DNA. PCR was performed to assemble the 16S rRNA gene (V3–V4 region), followed by sequencing in the San Diego Illumina Nova-PE250 (San Diego, CA, USA) at the Novogene Bioinformatics Institute. Sequence analysis was performed by QIIME (version 1.9.1., Flagstaff, AZ, USA), and the same operational classification units (OTUs) were assigned to series with ≥97% identity. Classification annotation was performed by the Ribosome Database Project (RDP) version 2.2 classifier. α-Diversity was analyzed using PAST3 (Olso, Norway). Non-metric multidimensional scaling (NMDS) and the unweighted pair group method with arithmetic mean (UPGMA) were calculated with the Novogene cloud platform (Beijing, China) on the basis of the weighted UniFrac distance. Following the linear discriminant analysis (LDA), the LDA effect size (LEfSe) analysis was conducted to identify the differential bacterial taxa from the level of the phylum to genus with two filters (*p* < 0.05 and LDA score > 4) (http://huttenhower.sph.harvard.edu/galaxy/) (accessed on 11 February 2022).

### 2.9. Real-Time Quantitative PCR (RT-qPCR)

RNA was extracted by Blotopped TRIzol reagent (Beijing, China), and then an RNA reverse transcription procedure was performed (TIANGEN, Beijing, China). A Bio-Rad CFX96 real-time PCR system (Hercules, CA, USA) was used to perform RT-qPCR analysis, accompanied by TIANGEN SYBER Green Supermix (Beijing, China). The mRNA expression was normalized using β-actin expression. The primer sequences are listed in Table 2.

### 2.10. Statistical Analysis

For all the data, the mean ± SEM was expressed and plotted as a graph with GraphPad Prism (version 9.0). One-way ANOVA was used to assess differences between groups and was considered statistically significant at *p* < 0.05.

## 3. Results

### 3.1. CA Has a Significant Preventive Effect against HFD-Induced Body Weight Gain in Mice

After 19 weeks of HFD feeding, significantly higher body weight and weight gain were observed in the mice in the HFD group compared with those in the Chow group, demonstrating that 19 weeks of HFD feeding was successful in inducing obesity. On the contrary, CA was significant in preventing HFD-induced obesity, which was evidenced by a decrease in the cumulative body weight gain in the mice (Table 3). In addition, the HFD and CA groups were comparable in food intake (Table 3); thus, the preventative effect of CA on HFD-induced obesity may not be attributed to appetite suppression.

### 3.2. CA Dramatically Decreases Visceral Fat Pad Weight, Attenuates Adipocyte Hypertrophy, and Improves Serum Lipid Profile in Mice

**Figure 2:**
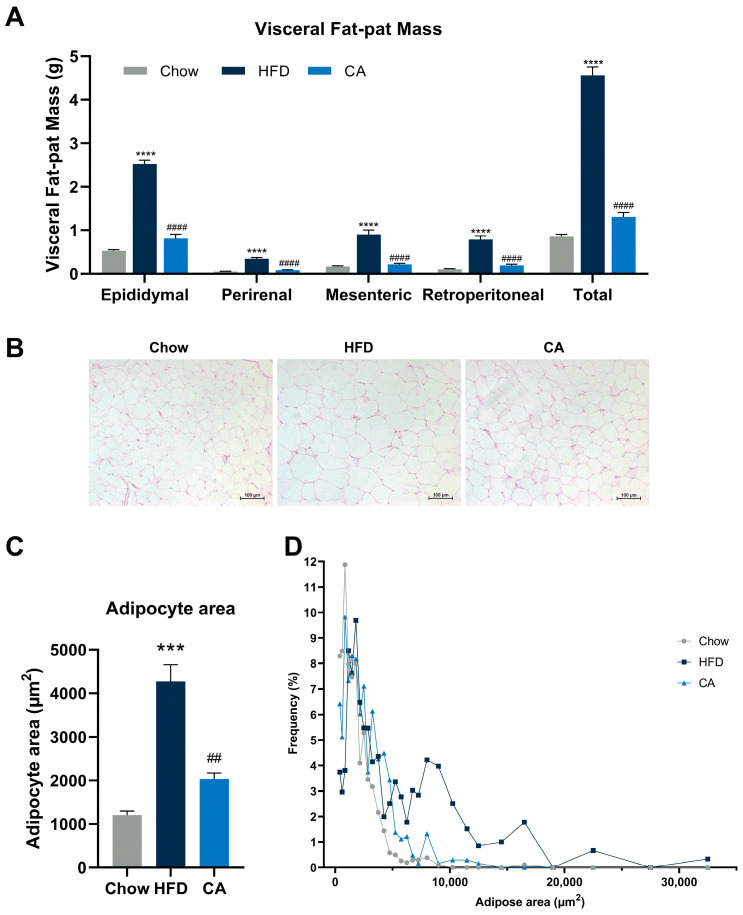
CA prevents HFD-induced adipocyte hypertrophy in mice. (**A**) The weight of visceral fat pads. (**B**) H&E-stained sections for eWAT, bar = 100 μm. (**C**) Mean cross-sectional areas of adipocytes. (**D**) Frequency distribution of adipocyte cross-sectional areas in eWAT. Data are expressed as means ± SEM (*n* = 8). **** *p* < 0.0001 vs. Chow group, *** *p* < 0.001 vs. Chow group, ^####^ *p* < 0.0001 vs. HFD group, ^##^ *p* < 0.01 vs. HFD group.

Consistent with the preventive effects of CA against body weight gain, the CA group had a significantly lower visceral fat-pad weight than the HFD group. (Figure 2A). Meanwhile, CA remarkably reduced adipocyte size in the epididymal white adipose tissue (eWAT) compared with that in the HFD group (2035 µm^2^ vs. 4275 µm^2^), as visualized by H&E-stained and quantified sections (Figure 2B–D). Serum chemistry parameters related to blood lipid levels were also measured, and CA supplementation produced a significant decrease in serum LDL-C, HDL-C, and TC (Table 4).

### 3.3. CA Improves Glucose Intolerance and Insulin Resistance in Mice

**Figure 3:**
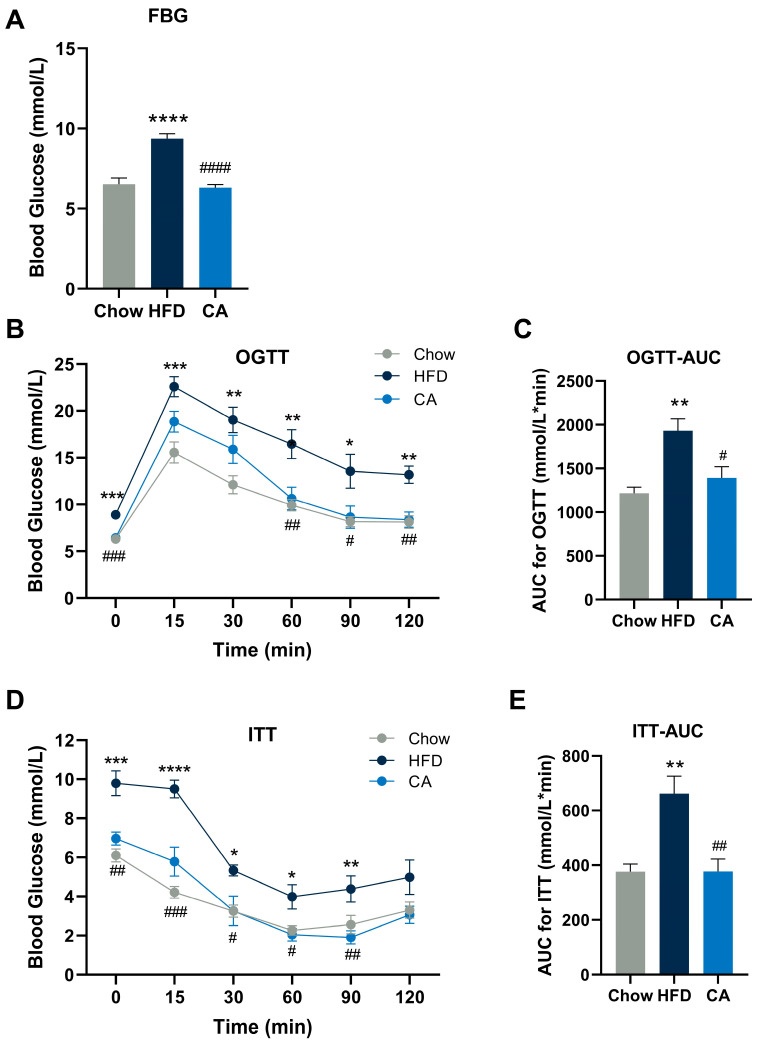
CA improves glucose intolerance and insulin resistance. (**A**) Fasting blood glucose (FBG) level. (**B**,**C**) Oral glucose tolerance test (OGTT) with its area under the curve (AUC). (**D**,**E**) Insulin tolerance test (ITT) with its AUC. Data are expressed as means ± SEM (*n* = 6). * *p* < 0.05 vs. Chow group, ** *p* < 0.01 vs. Chow group, *** *p* < 0.001 vs. Chow group, **** *p* < 0.0001 vs. Chow group, ^#^ *p* < 0.05 vs. HFD group, ^##^ *p* < 0.01 vs. HFD group, ^###^ *p* < 0.001 vs. HFD group, ^####^ *p* < 0.0001 vs. HFD group.

Common metabolic diseases associated with obesity also include impaired glucose metabolism; thus, whether CA could improve glucose metabolism was evaluated. In our study, compared with that in the HFD group, FBG decreased significantly in the CA group (Figure 3A). After glucose administration, the glucose levels at 60, 90, and 120 min were significantly lower in the CA group than in the HFD group (Figure 3B). The AUC_0–120min_ was calculated, and it indicated that glucose intolerance was improved with the supplementation of CA, as demonstrated by a significant decrease in the CA group in comparison with the HFD group (Figure 3C). Regarding insulin tolerance, after insulin injection, an evident decrease in plasma glucose concentrations at 15, 30, 60, and 90 min occurred in the CA group compared with those in the HFD group (Figure 3D). The AUC_0–120min_ was also remarkably reduced by CA supplementation, revealing that CA could improve insulin resistance (Figure 3E). In the OGTT and ITT tests, it was noteworthy that the CA group’s AUC levels were comparable to those of the Chow group.

### 3.4. CA Inhibits Hepatic Gluconeogenesis in HFD-Fed Mice

**Figure 4:**
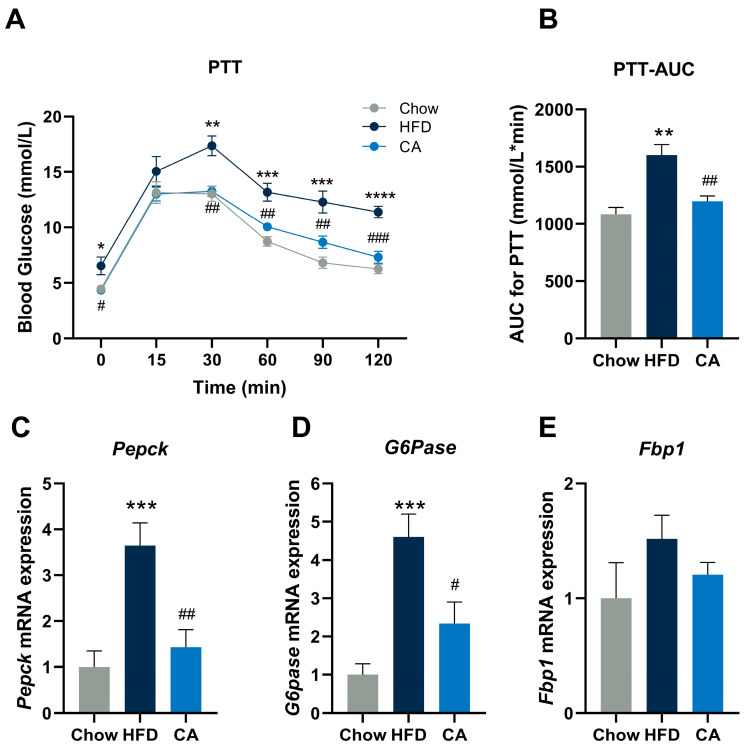
CA inhibits hepatic gluconeogenesis. (**A**,**B**) Pyruvate tolerance test (PTT) and its AUC. Data are expressed as means ± SEM (*n* = 6). (**C**–**E**) mRNA expression of genes involved in gluconeogenesis in the livers of mice (*n* = 6). *Pepck*, phosphoenolpyruvate carboxykinase; *G6Pase,* glucose-6-phosphatase, catalytic subunit; and *Fbp1*, fructose-1,6-bisphosphatase 1. * *p* < 0.05 vs. Chow group, ** *p* < 0.01 vs. Chow group, *** *p* < 0.001 vs. Chow group, **** *p* < 0.0001 vs. Chow group, ^#^ *p* < 0.05 vs. HFD group, ^##^ *p* < 0.01 vs. HFD group, ^###^ *p* < 0.001 vs. HFD group.

PTT was performed, and the HFD group exhibited an increase in gluconeogenesis compared with the Chow group. By contrast, in comparison with the HFD group, CA supplementation significantly inhibited the elevation of blood glucose levels at 30, 60, 90, and 120 min, and the AUC_0–120 min_ was significantly decreased (Figure 4A,B). Collectively, during the PTT test, it was indicated that CA could significantly inhibit gluconeogenesis in HFD-fed mice.

To further explore the mechanisms by which CA impacts glucose production, we measured the expression of genes participating in gluconeogenesis, a primary determinant of hepatic glucose production. As assessed by RT-qPCR, of the three key enzymes involved in gluconeogenesis—*Pepck*, *G6Pase*, and *Fbp1*—all were downregulated after CA supplementation, especially *Pepck* and *G6Pase* (Figure 4C–E).

### 3.5. CA Protectes against Hepatic Lipid Accumulation in Mice

**Figure 5:**
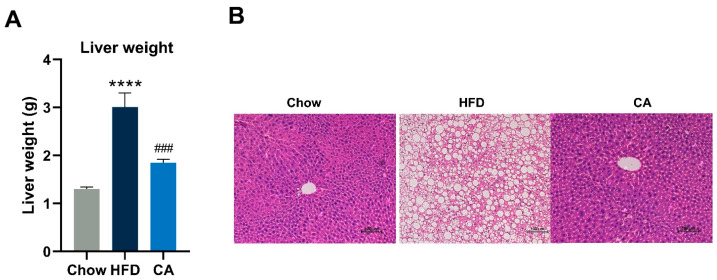
CA alleviates HFD-induced hepatic lipid accumulation. (**A**) Liver weight. (**B**) H&E-stained sections of liver. Bar = 100 μm. Data are expressed as means ± SEM (*n* = 8). **** *p* < 0.0001 vs. Chow group, ^###^ *p* < 0.001 vs. HFD group.

Obesity is commonly companioned by hepatic lipid accumulation, and we further examined whether CA could prevent hepatic lipid accumulation in mice. When compared with mice of the HFD group, liver weight was significantly reduced by CA supplementation (Figure 5A). In addition, CA showed a clear reduction in lipid accumulation and an orderly arrangement of hepatocytes, while mice in the HFD group showed a significant increase in neutral lipid droplets and an irregular arrangement of hepatocytes (Figure 5B). In addition, supplementation with CA significantly decreased the serum levels of ALT and AST, two markers of hepatotoxicity (Table 4).

### 3.6. Limited Effects of CA on the Modifying Gut Microbiota in HFD-Fed Mice

**Figure 6:**
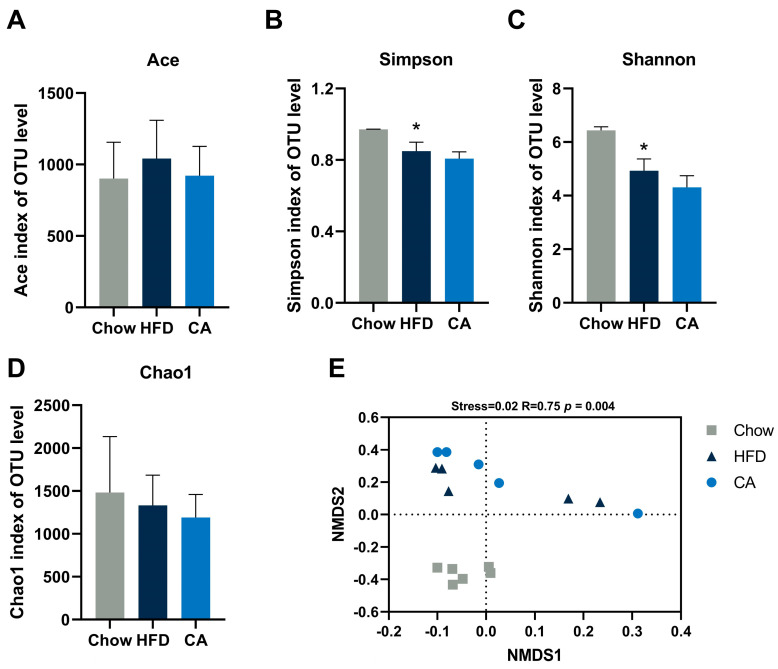
The effects of CA on the structure of gut microbiota. (**A**–**D**) Ace, Simpson, Shannon, and Chao1 indexes. (**E**) NMDS with weighted UniFrac distance; significant differences were assessed using adonis PERMANOVA. Data are expressed as means ± SEM (*n* = 5–6). * *p* < 0.05 vs. Chow group.

**Figure 7:**
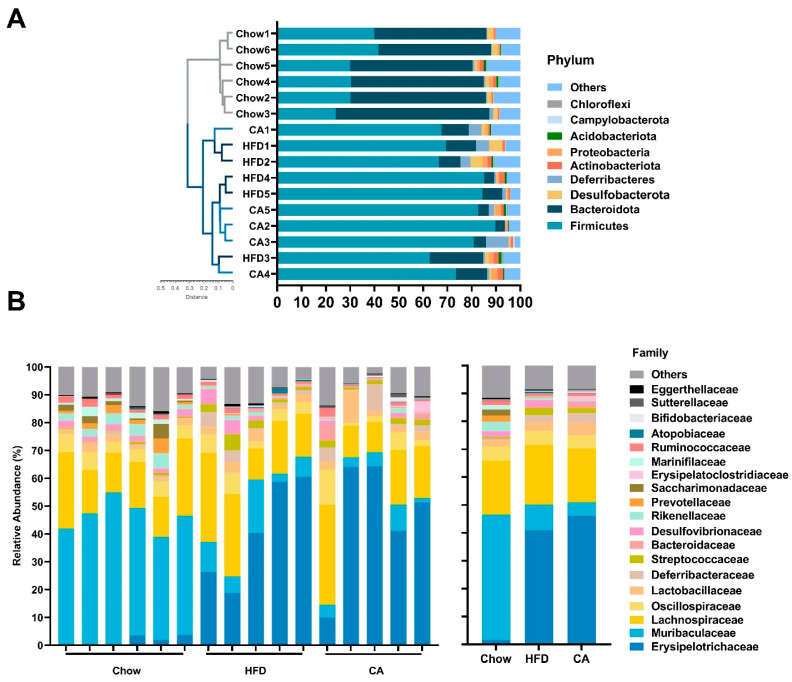
The composition of gut microbiota. (**A**) The relative abundance of the nine most abundant taxa at the phylum level; UPGMA with weighted UniFrac distance. (**B**) The relative abundance of the 19 most abundant taxa at the family level. Data are expressed as means (*n* = 5–6).

By sequencing the 16S rRNA gene, we measured the influence of CA supplementation on the structure and composition of the gut microbiota in mice. The indexes of α-diversity were analyzed at the OTU level and included ACE, Chao1, Simpson, and Shannon, with the former two indexes representing community richness and the latter two indexes representing community diversity. For the above-mentioned indexes, no considerable difference was found in the CA group compared with the HFD group (Figure 6A–D). Regarding β-diversity, the microbial communities of the CA group clustered together with the HFD group, while they were separated in the Chow group (Figure 6E). In addition, we calculated the composition of the gut microbiota in mice. Our results revealed that a similar composition at the phylum and family levels of the gut microbiota was observed in the CA and HFD groups, while the composition was significantly altered in the HFD group compared with the Chow group (Table 5). In addition, cluster analysis according to the UPGMA protocol indicated that no segregation existed between the CA and HFD groups, while clear segregation existed between the Chow and HFD groups (Figure 7A). Regarding the family level, the gut microbiota compositions were similar in the CA and the HFD groups, and the 17 most abundant taxa at the family level showed no significant difference between them (Figure 7B). An LEfSe analysis was also carried out, and only LDA scores over 4 were marked. The results revealed that dramatical taxonomical changes existed in the mice in the HFD group compared with those in the Chow group (Supplementary Figure S1A,B). Nevertheless, CA supplementation had a limited effect on modifying the gut microbiota, as revealed by the LEfSe analysis (Supplementary Figure S2A,B).

### 3.7. CA Alters the Expression of Metabolic Genes in eWAT

**Figure 8:**
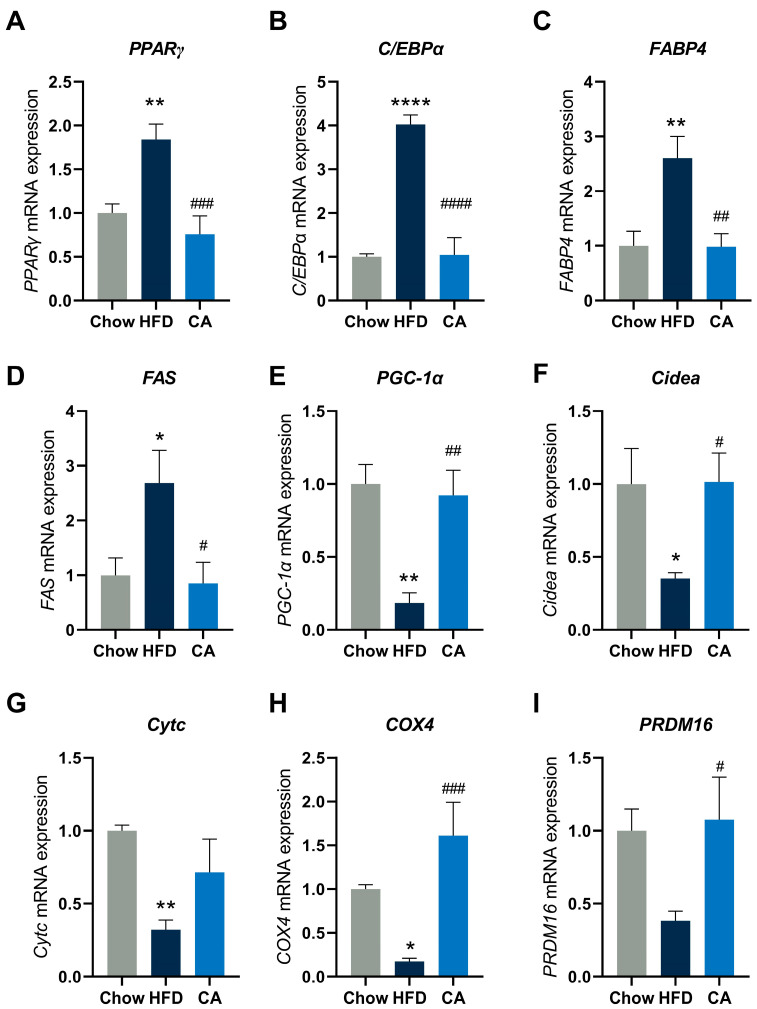
CA alters the expression of metabolic genes in eWAT. Data are expressed as means ± SEM (*n* = 6). The expression of (**A**) Peroxisome proliferator-activated receptor gamma (*PPARγ*), (**B**) CCAAT/enhancer binding-protein α (*C/EBPα*), (**C**) fatty acid-binding protein 4 (*FABP4*), (**D**) fatty acid synthase (*FAS*), (**E**) peroxisome proliferator-activated receptor gamma coactivator 1-alpha (*PGC-1α*), (**F**) cell death activator CIDE-A (*Cidea*), (**G**) cytochrome c (*Cytc*), (**H**) cytochrome c oxidase subunit 4 (*COX4*), and (**I**) PR domain containing 16 (*PRDM16*) in the epididymal adipose tissues of mice. * *p* < 0.05 vs. Chow group, ** *p* < 0.01 vs. Chow group, **** *p* < 0.0001 vs. Chow group, ^#^ *p* < 0.05 vs. HFD group, ^##^ *p* < 0.01 vs. HFD group, ^###^ *p* < 0.001 vs. HFD group, ^####^ *p* < 0.0001 vs. HFD group.

Considering that the WAT has an essential position in energy metabolism, we measured the mRNA expression of adipogenesis-associated genes in the eWAT in order to further evaluate the mechanism through which CA decreased the accumulation of lipid deposits. The *C/EBPα* and *PPAR-γ* are the major regulators of early adipogenesis but are also required to maintain the differentiated state of mature adipocytes. Our results confirmed that CA supplementation induced a marked decrease in *C/EBPα* and *PPAR-γ* expression compared with those in the HFD group (Figure 8A,B). We also observed a decrease in the expression of the main lipid synthesis molecules in the CA group, including *FABP4* and *FAS* (Figure 8C,D), which could respond to *PPAR-γ* and *C/EBPα*. This was consistent with our observation that CA reduced adipocyte hypertrophy (Figure 2). In addition, the results revealed that CA supplementation could also upregulate genes associated with the thermogenesis of eWAT, including *PGC-1α, PRDM16*, *Cidea*, *Cytc*, and *COX4* (Figure 8E–I). 

## 4. Discussion

In the present research, compared with mice in the HFD group, a decrease of approximately 30% in final body weight was observed in CA-treated mice (100 mg/kg) after 19 weeks; meanwhile, mice in the CA group had comparable body weights to those in the Chow group, indicating that CA has the potential to potently prevent obesity. Importantly, CA’s weight loss effect appeared to be unrelated to toxicity. During the 19-week study, neither death nor clinical signs of adverse treatment-related effects were observed. CA was reported to have a median acute lethal dose of 44,750 mg/kg in rats [27]. According to China’s acute toxicity dose classification standards (GB 15193.3-2014), it has been known to be practically non-toxic. Herein, no hepatotoxicity (Table 4), abnormalities, pathological histological changes, or specific injury manifestations related to the subjects were found on their anatomy. Nonetheless, comprehensive safety studies are needed before the pharmaceutical use of CA.

Excessive consumption of an HFD has doubtlessly contributed to the prevalence of obesity [28]. A multitude of studies has recently indicated that modifications in the gut microbiota due to HFDs are related to the obesity epidemic [29,30]. In the gut microbiota, the main dominant phyla are Bacteroides and Firmicutes [31], and an increase in Firmicutes and a decrease in Bacteroidetes (i.e., increased F/B ratio) are generally accompanied by HFD consumption [32], which is similar to our results (Table 5). Nevertheless, contrary to this, a number of studies have observed that this parameter was not altered to any degree and have even found that obese animals have a decreased F/B ratio [33,34,35]. On the one hand, these differences may be due to several factors that could affect the gut microbiota, in terms of the genetic background of the host, age, sex, and time of gut transport [36,37]. On the other hand, a point that is easily ignored but indeed exists is that different methodologies could also affect the results of the gut microbiota composition. Allali et al. reported a difference in the determination of microbial diversity and species richness between sequencing platforms and library preparation protocols [38].

Regarding the changes in the gut microbiota between the CA and the HFD groups, no significant changes in α-diversity or β-diversity were observed, and the gut microbiota compositions were similar in the two groups. In detail, compared with mice fed with HFD, CA-treated mice exhibited no significant change in the relative abundance of the nine most abundant phylum-level taxa, and they did not show significant changes in the 17 most abundant family-level taxa. These results indicated that CA may ameliorate HFD-induced obesity independently of the gut microbiota. Similarly, some dietary components and natural products exert beneficial effects without altering the gut microbiota. Trans-resveratrol reduced both weight gain and serum insulin levels in mice while scarcely modifying the profile of the gut bacteria [39]. Metformin reduces adiposity and/or low inflammation per se to improve metabolic syndrome instead of interacting directly with the gut microbiota [40]. Nevertheless, certain plant components have been reported to have beneficial effects associated with the gut microbial composition in obesity models, such as xanthohumol derivatives [41,42] and epigallocatechin-3-gallate [43]. In summary, our results initially suggest that CA may have a limited effect on modifying the gut microbiota, and the effects of different natural products on the gut microbiota are probably related to the properties of the substances themselves.

Excessive accumulation of WAT is a defining characteristic of obesity, and the remodeling process of WAT includes the proliferation of adipocytes (defined as adipogenesis) [44]. The adipogenesis process is critically regulated by various signaling molecules and several key adipose transcription factors, particularly *PPARγ* and *C/EBPα*, the activation of which is essential for adipocyte differentiation [45]. In addition, they contribute to encoding lipid synthesis-related molecules, including *FABP4* and *FAS*, which could promote lipogenic binding and lipid storage [46]. Our results showed that the *PPARγ*, *C/EBPα*, *FABP4,* and *FAS* genes were significantly downregulated in CA-treated mice (Figure 8), suggesting that CA may have the ability to reduce lipid accumulation and further alleviate obesity by restraining adipogenesis and lipid synthesis. In addition, CA enhanced the expression of *PGC-1α*, *Cidea*, *PRDM16*, *COX4*, and *Cytc* in the eWAT after CA supplementation (Figure 8). These genes participate in numerous biological functions, and their increased expression plays a role in promoting thermogenesis [47]. Altogether, these data suggest that CA could primarily downregulate genes involved in adipogenesis and lipid synthesis and upregulate genes related to thermogenesis; thus, the effect of CA on lowering lipid levels and alleviating obesity and its related metabolic syndrome was probably associated with the alteration of these metabolism-related genes.

Obesity is often followed by hepatic lipid accumulation resulting from an increased supply of lipids to the liver as lipids from adipose tissue spill over into the circulation [48,49]. In the hepatocytes, such an excessive supply contributes to the aggregation of lipid droplets. In addition, obesity leads to systemic insulin resistance, one of the fundamental aspects of the etiology of type 2 diabetes [50]. According to our results, CA supplementation reduced obesity, alleviated hepatic lipid accumulation, and inhibited hepatic gluconeogenesis in mice fed an HFD. Moderate weight loss was reported to reverse the accumulation of hepatic lipids and insulin resistance and normalize hepatic glucose production by reducing gluconeogenesis [51]. Hence, in CA-treated mice, the amelioration in hepatic lipid accumulation and insulin resistance observed may be a secondary event following a reduction in adiposity. There is a need to evaluate the mechanism by which CA improves hepatic lipid accumulation and insulin resistance in the future.

## 5. Conclusions

Overall, our research indicated that CA significantly reduced body weight gain, decreased the weight of visceral fat pads, and prevented adipocyte hypertrophy in HFD-fed mice. The HFD-induced hepatic lipid accumulation and impaired glucose metabolism were also ameliorated in mice in the CA group. The above beneficial effects of CA on obesity and obesity-related metabolic syndrome may be independent of the gut microbiota and rather associated with its regulation of metabolism-related gene expression. In a word, CA has the potential to be a prospective dietary component for obesity prevention.

## Figures and Tables

**Table 1:** Experimental diet composition (g/kg).

Ingredients	HFD Group	CA Group
Casein	200	200
DL-Methionine	3	3
Corn starch	111	110
Sucrose	370	370
Cellulose	50	50
Corn oil	30	30
Lard	170	170
AIN-76A Mineral mixture	42	42
AIN-76A Vitamin mixture	12	12
Choline bitartrate	2	2
Cholesterol	10	10
Cedryl acetate	-	1
tert-Butylhydroquinone	0.04	0.04
Fat, % kJ	40	40

**Table 2:** Primer sequences used for RT-qPCR.

Target Genes	Forward Primer 5′–3′	Reverse Primer 5′–3′
*C/EBPα*	TCAGCTTACAACAGGCCAGG	ACACAAGGCTAATGGTCCCC
*Cidea*	GGAATCTGCTGAGGTTTATG	ATCCCACAGCCTATAACAGA
*COX4*	GTACCGCATCCAGTTTAACGA	CCATACACATAGCTCTTCTCCCA
*Cytc*	ACACTGTGGAAAAGGGAGGC	GCACTGGTTAACCCAAGCAA
*FABP4*	CATGCGACAAAGGCAGAAAT	GTTACAAGGCAAGGAAGGGC
*FAS*	TTGCTGGCACTACAGAATGC	AACAGCCTCAGAGCGACAAT
*Fbp1*	GTGTCAACTGCTTCATGCTG	GAGATACTCATTGATGGCAGGG
*G6pase*	CGACTCGCTATCTCCAAGTGA	GTTGAACCAGTCTCCGACCA
*Pepck*	CCATCACCTCCTGGAAGAACA	ACCCTCAATGGGTACTCCTTCTG
*PGC-1α*	AAATCTGCGGGATGATGGA	GTTTCGTTCGACCTGCGTAA
*PPARγ*	TTCGGAATCAGCTCTGTGGA	CCATTGGGTCAGCTCTTGTG
*PRDM16*	CCACCAGCGAGGACTTCAC	GGAGGACTCTCGTAGCTCGAA
*β-actin*	GGCTGTATTCCCCTCCATCG	CCAGTTGGTAACAATGCCATGT

**Table 3:** Effects of CA on body weight and food intake of HFD-fed mice.

	Chow	HFD	CA
Initial body weight (g)	23.85 ± 0.39	24.93 ± 0.34	24.75 ± 0.39
Final body weight (g)	29.84 ± 0.54	46.61 ± 1.39 ****	33.68 ± 0.92 ^####^
Body weight gain (g)	5.99 ± 0.41	21.69 ± 1.41 ****	8.93 ± 0.90 ^####^
Food intake (g/day)	4.09 ± 0.02	3.32 ± 0.10 **	3.20 ± 0.10

Data are expressed as means ± SEM, *n* = 8. **** *p* < 0.0001 vs. Chow group, ** *p* < 0.01 vs. Chow group, ^####^ *p* < 0.0001 vs. HFD group.

**Table 4:** Serum biochemical analysis.

	Chow	HFD	CA
LDL-C (mmol/L)	0.17 ± 0.01	1.49 ± 0.25 ****	0.72 ± 0.11 ^##^
HDL-C (mmol/L)	3.46 ± 0.18	6.57 ± 0.24 ****	4.83 ± 0.51 ^##^
TC (mmol/L)	3.06 ± 0.18	8.57 ± 0.74 ****	4.70 ± 0.35 ^####^
ALT (U/L)	43.1 ± 4.0	237.3 ± 60.4 **	61.4 ± 11.8 ^##^
AST (mmol/L)	369 ± 28	568 ± 67 *	429 ± 50

Data are presented as means ± SEM, *n* = 6. ALT, alanine aminotransferase; AST, aspartate aminotransferase; LDL-C, low-density lipoprotein cholesterol; HDL-C, high-density lipoprotein cholesterol; TC, total cholesterol. * *p* < 0.05 vs. Chow group, ** *p* < 0.01 vs. Chow group, **** *p* < 0.0001 vs. Chow group, ^##^ *p* < 0.01 vs. HFD group, ^####^ *p* < 0.0001 vs. HFD group.

**Table 5:** The abundance of gut microbiota at the phylum level and family level.

	Chow	HFD	CA
Phylum			
Firmicutes	32.67%	73.61% ****	78.82%
Bacteroidota	52.75%	11.08% ****	7.49%
Deferribacteres	0.63%	2.54%	3.56%
Desulfobacterota	1.81%	2.58%	0.72%
Proteobacteria	0.76%	1.09%	1.49%
Actinobacteriota	0.84%	1.54%	1.00%
Acidobacteriota	0.35%	0.53%	0.41%
Campylobacterota	0.08%	0.10%	0.21%
Chloroflexi	0.12%	0.2%	0.18%
Ratio of F/B	0.64	9.50 *	13.92
Family			
Erysipelotrichaceae	1.55%	40.92% **	46.17%
Muribaculaceae	44.91%	9.22% ****	4.79%
Lachnospiraceae	19.34%	21.39%	19.25%
Oscillospiraceae	5.29%	5.11%	4.88%
Lactobacillaceae	2.1%	2.99%	4.42%
Deferribacteraceae	0.63%	2.54%	3.56%
Streptococcaceae	0.38%	2.62% *	1.68%
Bacteroidaceae	0.45%	0.26%	1.77%
Desulfovibrionaceae	1.78%	2.52%	0.66%
Rikenellaceae	3.42%	0.96% **	0.55%
Prevotellaceae	2.24%	0.26% *	0.15%
Saccharimonadaceae	2.08%	0.04% *	0.01%
Erysipelatoclostridiaceae	0.08%	0.12%	1.01%
Marinifilaceae	1.47%	0.03% **	0.01%
Ruminococcaceae	1.88%	1.08%	1.15%
Atopobiaceae	0.08%	0.54%	0.11%
Bifidobacteriaceae	0.04%	0.32%	0.50%
Sutterellaceae	0.18%	0.07%	0.64% ^##^
Eggerthellaceae	0.43%	0.41%	0.17%

Data are presented as mean (*n* = 5–6). * *p* < 0.05 vs. Chow group, ** *p* < 0.01 vs. Chow group, **** *p* < 0.0001 vs. Chow group, ^##^ *p* < 0.01 vs. HFD group.

## Data Availability

The data that support the findings of this study are available from the corresponding author upon reasonable request.

## Supplementary Materials

The following supporting information can be downloaded at: https://www.mdpi.com/article/10.3390/nu15040980/s1, Table S1. Composition of high-fat diet and standard chow diet (Rodent Diet 1025, HFK Bioscience). Figure S1. (A) Linear discriminant analysis (LDA) with effect size (LEfSe) in Chow and HFD groups. (B) Cladogram obtained from LEfSe analysis of differentially abundant taxa at the phylum, class, order, family, and genus levels. Figure S2. (A) LEfSe in HFD and CA groups. (B) Cladogram obtained from LEfSe analysis of differentially abundant taxa at the phylum, class, order, family, and genus levels.
