# Supplementary material for: Dietary Supplementation of Cedryl Acetate Ameliorates Adiposity and Improves Glucose Homeostasis in High-Fat Diet-Fed Mice
Source: Nutrients. 2023 Feb 16;15(4):980. doi: 10.3390/nu15040980 (PMC9967006; doi:10.3390/nu15040980)
Supplement: Supplementary file 1 [file nutrients-15-00980-s001.zip › nutrients-2192548-supplementary.pdf]

nutrients MDPI

Supplementary Material

Article

Dietary Supplementation of Cedryl Acetate Ameliorates Adiposity and Improves Glucose Homeostasis in High-Fat Diet-Fed Mice

Jingya Guo [1], Mengjie Li [1], Yuhan Zhao [1], Seong-Gook Kang [2], Kunlun Huang [1,3,4] and Tao Tong [1,3,4,*]

Table S1. Composition of high-fat diet and standard chow diet (Rodent Diet 1025, HFK Bioscience).

	HFD (4.6 Kcal/g)	Chow (3.42 Kcal/g)
**Nutrient Information**	**% kcal from**	**% kcal from**
Protein	18.0	22.5
Carbohydrate	42.0	65.4
Fat	40.0	12.1
**Formula**	**g/kg**	**Representative Ingredients**
Casein	200	Soybean meal
DL-Methionine	3	Lysine
Corn starch	111	Ground corn
Sucrose	370	Wheat flour
Cellulose	50	Wheat middlings
Corn oil	30	Fish meal
Lard	170	Soybean oil
Cholesterol	10	
Mineral mixture	42	Mineral mixture, Calcium carbonate, Calcium bicarbonate
Vitamin mixture	12	Vitamin mixture
Choline bitartrate	2	Choline chloride
tert-Butylhydroquinone	0.04	/

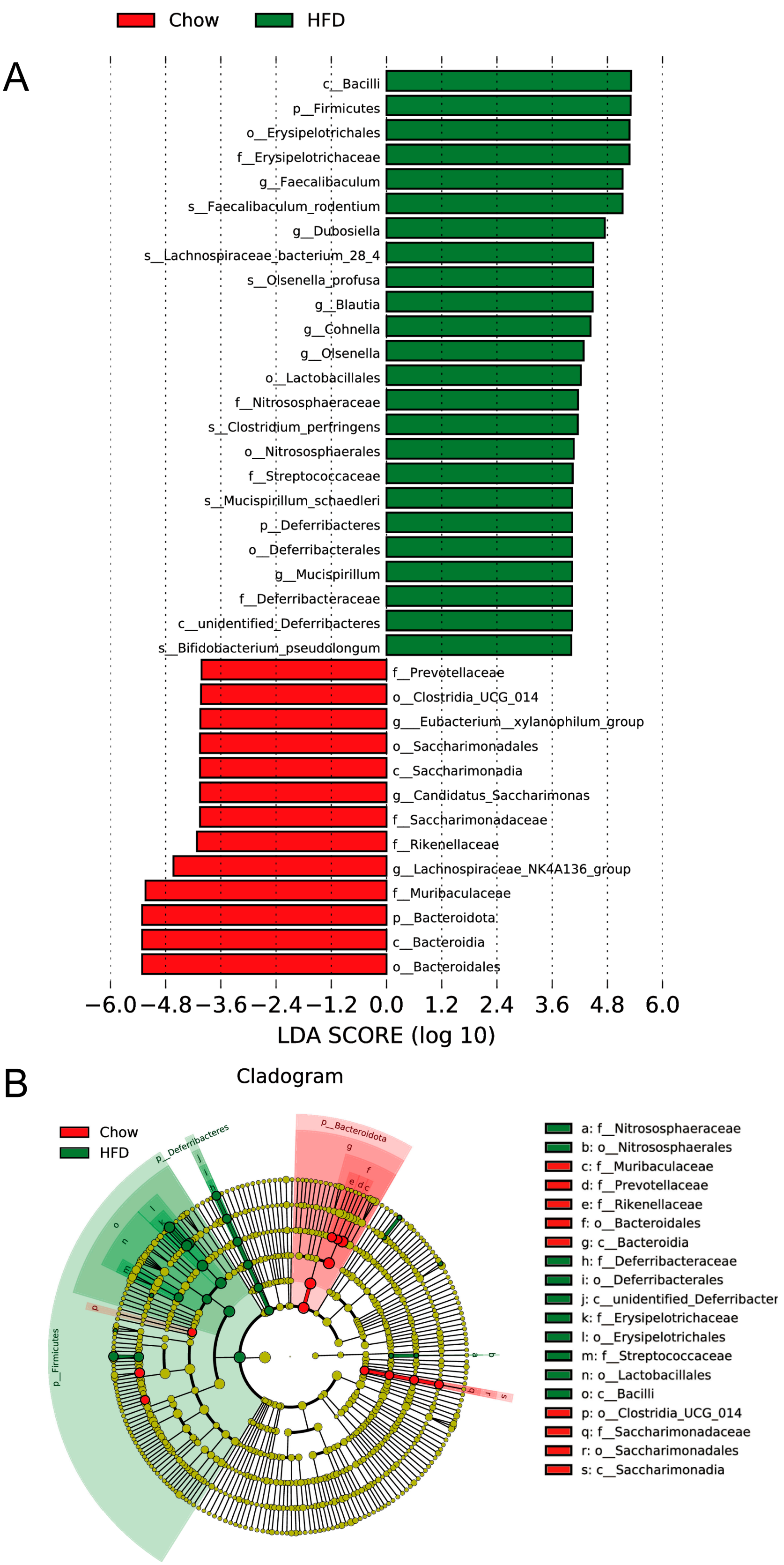

Figure S1. (**A**) Linear discriminant analysis (LDA) with effect size (LEfSe) in Chow and HFD groups. (**B**) Cladogram obtained from LEfSe analysis of differentially abundant taxa at the phylum, class, order, family, and genus level.

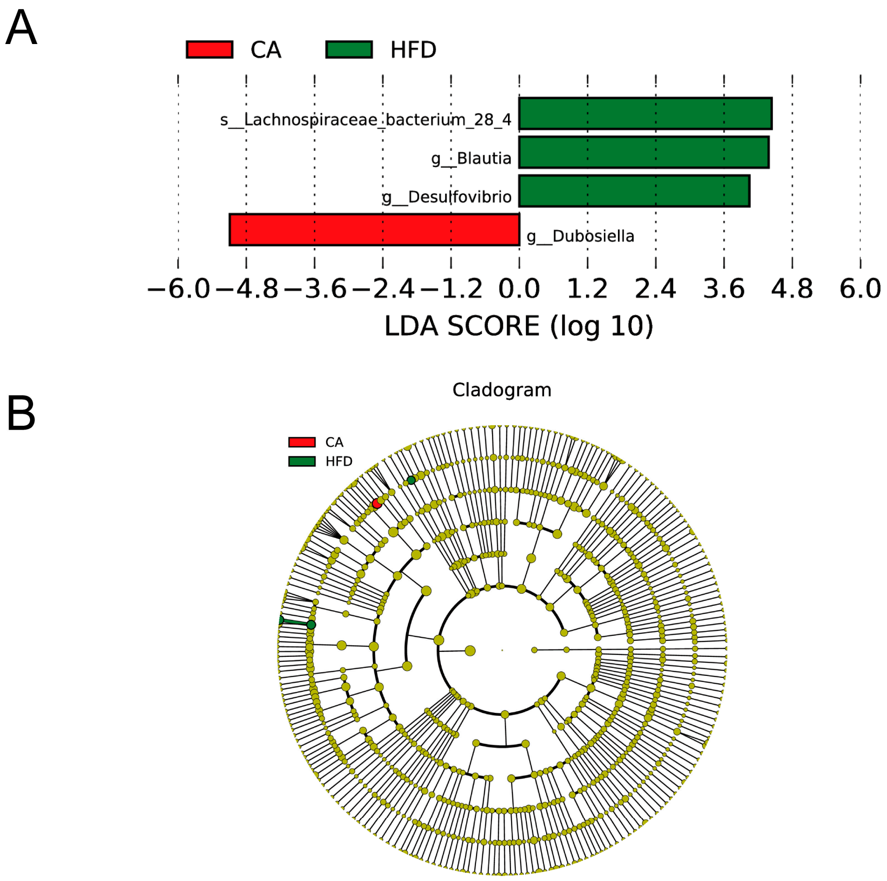

Figure S2. (**A**) LEfSe in HFD and CA groups. (**B**) Cladogram obtained from LEfSe analysis of differentially abundant taxa at the phylum, class, order, family, and genus level.
